# Vitamin D supplementation in patients with diabetes mellitus type 2 on different therapeutic regimens: a one-year prospective study

**DOI:** 10.1186/1475-2840-12-113

**Published:** 2013-08-07

**Authors:** Khalid M Alkharfy, Nasser M Al-Daghri, Shaun B Sabico, Abdulaziz Al-Othman, Osama Moharram, Majed S Alokail, Yousef Al-Saleh, Sudhesh Kumar, George P Chrousos

**Affiliations:** 1 Department of Clinical Pharmacy, College of Pharmacy, King Saud University, PO Box 2457, 11451, Riyadh, Saudi Arabia; 2 Biochemistry Department, College of Science, King Saud University, PO Box 2455, 11451, Riyadh, Saudi Arabia; 3 Biomarkers Research Program, King Saud University, 11451, Riyadh, Saudi Arabia; 4 Prince Mutaib Chair for Biomarkers of Osteoporosis, King Saud University, 11451, Riyadh, Saudi Arabia; 5 College of Applied Medical Sciences, King Saud University, 11451, Riyadh, Saudi Arabia; 6 King Abdulaziz University Hospital, King Saud University, 11451, Riyadh, Saudi Arabia; 7 King Abdulaziz Medical City, 11426, Riyadh, Saudi Arabia; 8 Division of Metabolic and Vascular Health, Clinical Sciences Research Institute, University Hospitals Coventry and Warwickshire Trust, Walsgrave, CV2 2DX, Coventry, UK; 9 First Department of Pediatrics, Athens University Medical School, 11527, Athens, Greece

**Keywords:** Vitamin D, Vitamin D supplementation, Diabetes mellitus, Anti-diabetes therapies

## Abstract

**Background:**

Little or no research has determined the effect of vitamin D3 supplementation in conjunction with pharmacological and non-pharmacological approaches in the diabetes mellitus type 2 (DMT2) patients. The objective of this study was to determine the effect of vitamin D3 supplementation in a cohort of Saudi DMT2 population on diet, insulin and/or different oral hypoglycemic agents and compare them with a non-DMT2 control cohort.

**Methods:**

A total of 499 randomly selected Saudi subjects divided into 8 groups [Non-DMT2 Control = 151; Rosiglitazone alone = 49; Diet = 15; Insulin alone = 55; Insulin + Orals = 12; Metformin alone = 121; Oral agents combination = 37; Sulphonylurea alone = 59] were included in this 12-month interventional study. All DMT2 patients were given 2000 IU vitamin D3 daily, while the control group received none but were advised to increase sun exposure. Anthropometrics, glucose, lipid profile and 25-hydroxyvitamin D (25-OHVitD) were measured at baseline, 6 and 12 months.

**Results:**

Circulating 25-OHVitD concentrations improved in all patient groups. The metformin group showed the highest change in circulating vitamin D levels both at 6 months (62.6%) and 12 months (50.6%) as compared to baseline (p < 0.001). No significant changes were observed in the BMI and glucose in any of the DMT2 groups. In contrast, the insulin + oral agents group showed more significant improvements in the metabolic profile, which included triglycerides and total cholesterol, as well as systolic blood pressure and HDL-cholesterol in males. Also, significant decreases in triglycerides were observed in the rosiglitazone and insulin + oral hypoglycemic agent groups both at 6 and 12 months of supplementation (both p-values <0.001).

**Conclusion:**

While in all DMT2 groups circulating levels of 25-OHVitD increased after supplementation, in DMT2 patients on insulin in combination with other drugs benefitted the most in improving cardiovascular risk. Metformin improves 25-hydroxyvitamin D levels but did not seem to confer other added cardiometabolic benefits.

## Background

Vitamin D deficiency has been a global pandemic for a while [1,2], yet the level of attention given by the scientific and clinical community was only recently stimulated, primarily because of the pleiotropic effects of this hormone outside the skeletal system. Vitamin D deficiency has been consistently associated with hypertension, diabetes mellitus, cardiovascular disease, stroke, multiple sclerosis, inflammatory bowel disease, osteoporosis, periodontal disease, macular degeneration, mental illness, propensity to fall, chronic pain and various cancers [3]. In the Kingdom of Saudi Arabia (KSA), as well as neighboring countries in the Middle East and North Africa, where there is a recent surge in the incidence of osteoporosis, major risk factors included evidence that points to an overwhelming prevalence of vitamin D deficiency [4].

Several interventional studies have been conducted in response to the dilemma mentioned above. We previously observed modest benefits conferred by vitamin D correction in deficient populations through non-pharmacologic means among adults [5,6]. We also documented the benefits of vitamin D supplementation among patients with diabetes mellitus type 2 (DMT2) [7], another chronic non-communicable disease highly prevalent in KSA [8]. In all previous studies mentioned, the favorable effects of improved vitamin D status are most evident in the lipid profile of subjects, reaffirming the hypothesis that vitamin D deficiency contributes to the pathogenesis of atherogenic dyslipidemia [9].

Little or no research has determined the effect of vitamin D3 supplementation when given in conjunction with existing non-pharmacological and pharmacological approaches to the DMT2 population. Hence, this study primarily determined the effect of vitamin D3 supplementation in a cohort of Saudi DMT2 population on different therapeutic regimens and compared them with a non-DMT2, non-vitamin D supplemented control cohort.

## Methods

### Site and duration of the study

This is a multi-center, interventional study conducted at 5 primary health care out-patient clinics (Al-Fawaz, Al-Nasim, Al-Marwah, Al-Badia and Gubeirah) and one tertiary hospital (King Abdulaziz University Hospital) in the central region of Riyadh, KSA. Ethics approval was obtained from the Ethics Committee of the College of Medicine Research Center at King Saud University, Riyadh, KSA.

### Subjects

A total of 760 adult Saudi patients with or without DMT2 were initially recruited to take part in this interventional study. These subjects were recruited as part of the on-going Biomarker Screening Project of King Saud University in collaboration with the Ministry of Health which began in 2008. In brief, this project is connected with the different primary health care centers in Riyadh and aims to recruit randomly selected Saudis aged 1–85 for population and epidemiological studies on a nationwide scale. Out of the 760 subjects, 261 dropped out for various reasons (non-compliance, change of medication, lost to follow up, etc.). The final count, 348 adult Saudi patients (195 males and 153 females) with controlled DMT2 aged 21 years and above but not exceeding 60 years of age, and another 151 matched non-diabetic Saudi adults (72 males and 79 females) were the only subjects who were able to finish the intervention study. Exclusion criteria were the following: Patients taking mineral oil products, using antacids regularly, taking cortisone or other steroids, under diuretics, taking weight-loss drugs, under phenobarbital and phenytoin medications, having liver problems, gallbladder disease or gastrointestinal disorders and taking daily multivitamins including calcium. Calcium metabolism abnormalities such as evidence of metabolic disease (Paget’s disease or osteomalacia), hyperparathyroidism, renal stone disease, and abnormal levels of calcium, phosphorous and alkaline phosphatase were also excluded.

Subjects were divided into 8 groups based on their existing diabetic therapy: (A) Non-DMT2 Control (B) Rosiglitazone (C) Diet (D) Insulin (E) Insulin + Orals (F) Metformin (G) Oral agents-combination (those who are not on monotherapy aside from insulin + orals) and (H) Sulphonylureas. Each DMT2 group was given vitamin D supplements (2000 IU) in tablet form (Vigantoletten; Merck Pharma, Germany) daily for 12 months during which all laboratory parameters were repeated. The non-DMT2 control group were not given vitamin D supplements but instead were given advise to increase sun exposure (minimum body parts exposed should include face, neck, hands and arms, or at least 26% of the body surface area) and daylight outdoor activity during the entire study. All subjects were provided with a general questionnaire which includes thorough past and present medical history and detailed medication information. Subjects were required to submit written informed consents prior to being included in the study. None of the subjects included in the analysis changed their medication regimen during the entire study period.

### Anthropometrics

All anthropometric parameters were measured while the subject was standing erect and barefoot. The hips were measured using a standardized non-stretchable fiber measuring tape. It was then taken as the greatest circumference at the level of greater trochanters (the widest portion of the hip) on both sides. The waist girth was measured as the smallest horizontal girth between the costal margins and the iliac crests at minimal respiration. Waist-hip ratio was calculated using the formula waist (cm) divided by hips (cm). Height and weight were determined using standardized conventional methods. Body mass index was calculated using the formula: weight in kilograms (kg) divided by height in squared meters (m^2^). A standardized mercury sphygmomanometer was used to take the blood pressure of each participant 30 minutes after complete rest.

### Laboratory parameters

Subjects were required to submit overnight fasting blood samples utilized for the different metabolic parameters. Fasting blood glucose and lipid profile were determined using routine laboratory procedures (Konelab, Finland). 25-hydroxyvitamin D (25-OHVitD) level was measured using an enzyme-linked immunosorbent assay (ELISA) (IDS Ltd., Boldon Colliery, Tyne & Wear, UK). The inter- and intra-assay variabilities were 5.3% and 4.6%. All measurements were done in a DEQAS- (Vitamin D External Quality Assessment Scheme) participating laboratory, the Biomarkers Research Program (BRP) of King Saud University, Riyadh, KSA.

### Statistical analysis

All statistical analyses were done using SAS (Statistical Analysis System) software version 9.1.3; (SAS institute, Cary, NC, USA). Non–Gaussian variables were logarithmically transformed. Data was quantified by least square means and corresponding standard error. To compare differences between treatment groups in outcomes over time, General Linear models (PROC GLM procedure) was used. Statistically significant follow up differences or effects from baseline were determined using Bonferroni multiple comparison test. Level of significance was set at p ≤ 0.05.

## Results

### Vitamin D

**Figure 1:**
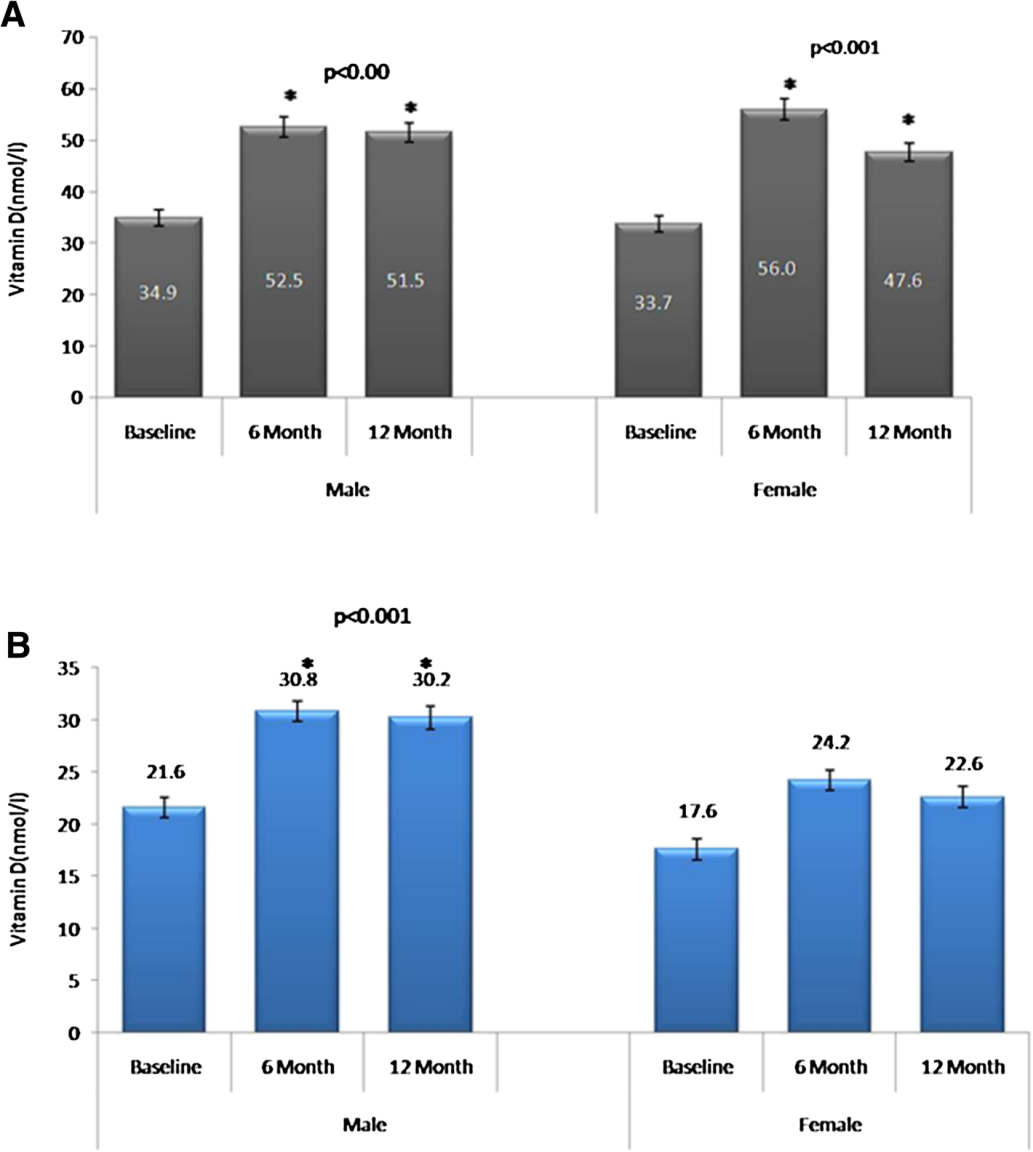
**Circulating 25-Hydroxyvitamin D Levels in A.** DMT2 Males and Females and **B.** Non-DMT2 Control Males and Females.

With the exception of the diet and oral agents-combination group, all other groups showed a significant increase in serum 25-OHVitD levels as compared with mean baseline status (Table 1). Furthermore, this improvement in 25-OHVitD status was most pronounced after 6 months supplementation, with the 12-month follow-up slightly lower than the 6-month follow-up, but still significantly higher than baseline. This observation remained consistent even after stratifying the groups according to gender. The rosiglitazone and diet groups in females failed to achieve significance possibly because of the small sample size (Table 2). A comparison of 25-OHVitD levels between control and DMT2 groups showed higher baseline 25-OHVitD in males (both control and DMT2) than females (Figure 1).

**Table 1:** Group comparisons of metabolic parameters at different time points

	**Control**	**Rosiglitazone**	**Diet**	**Insulin**	**Insulin + Orals**	**Metformin**	**Oral agents combination**	**Sulphonylurea**
N	151	49	15	55	12	121	37	59
Gender (M/F)	72/79	48/1	4/11	30/25	6/6	65/56	16/21	26/33
Obesity (%)	41.3	51.1	66.7	73.8	66.7	57.3	79.4	48.9
Hypertension (%)	21.9	38.8	26.7	29.1	29.1	32.2	25.0	27.1
Dyslipidemia (%)	0.7	16.3	6.7	14.5	8.3	12.4	2.7	10.2
**25-Hydroxyvitamin D (nmol/l)**								
Baseline	19.4 ± 1.0	33.3 ± 1.0	24.4 ± 1.1	32.2 ± 1.0	34.1 ± 1.1	34.8 ± 1.0	34.6 ± 1.1	38.3 ± 1.0
6 months	27.4 ± 1.0*	47.8 ± 1.1*	34.3 ± 1.2	55.7 ± 1.1*	55.8 ± 1.1*	62.4 ± 1.1*	46.0 ± 1.1	51.2 ± 1.1*
12 months	25.8 ± 1.1*	49.9 ± 1.2*	26.7 ± 1.3	46.1 ± 1.1*	48.1 ± 1.2*	55.3 ± 1.1*	46.8 ± 1.1	46.8 ± 1.1*
% Change at 6 months ^C^	34.3 (12.0, 56.6)^a^	36 (10.2, 61.8)^b^	34.1 (−9.3, 72.3)	54.3 (25.0, 83.7)^b^	49.1 (−3.2, 68.3)	62.6 (39.1, 86.1) ^a^	28.5 (−7.3, 64.4)	28.8 (2.2, 55.4)^b^
% Change at 12 months ^C^	28.6 (6.0, 51.4) ^a^	40 (3.2, 77.3)^a^	9.2 (−12.8,65.3)	35.6 (3.2, 68.0)^a^	34.3 (−2.1, 69.7)	50.6 (23.4, 77.9) ^a^	30.2 (−14.0,74.4)	19.8 (−13.2, 52.9)^a^
*P*-value (groups) < 0.001; *P*-value (time) < 0.001; *P*-Value (interaction) = 0.04								
**Systolic BP (mmHg)**								
Baseline	115.6 ± 1.1	129.0 ± 1.0	124.2 ± 1.0	126.5 ± 1.0	129.1 ± 1.0	126.1 ± 1.0	134.1 ± 1.1	126.5 ± 1.0
6 months	113.3 ± 1.1	130.0 ± 1.0	124.3 ± 1.0	123.5 ± 1.0	132.5 ± 1.0	124.8 ± 1.0	131.1 ± 1.0	129.7 ± 1.0
12 months	115.4 ± 1.1	130.9 ± 1.0	130.0 ± 1.1	128.1 ± 1.0	130.5 ± 1.0	127.4 ± 1.1	127.7 ± 1.0	127.9 ± 1.0
% Change at 6 months ^C^	−2.4 (−8.8, 3.8)	1.4 (−5.8, 8.7)	0.0	−2.4 (−10.4, 5.5)	2.1 (−12.2, 16.6)	−1.0 (−6.3,4.3)	−2.2 (−11.6, 7.1)	2.5 (−4.9, 9.9)
% Change at 12 months ^C^	0.2 (−7.0, 6.7)	0.9 (−8.1, 10.0)	4.7 (−24.8, 34.2)	1.1 (−8.8, 11.1)	0.6 (−16.0, 17.3)	1.0 (−5.2, 7.4)	−4.8 (−16.5, 6.7)	1.1 (−7.8, 10.0)
*P*-value (groups) < 0.001; *P*-value (time) = 0.51; *P*-Value (interaction) = 0.71								
**Diastolic BP (mmHg)**								
Baseline	74.5 ± 1.0	71.5 ± 1.0	77.4 ± 1.2	78.2 ± 1.0	75.6 ± 1.0	80.5 ± 1.2	77.5 ± 1.0	79.8 ± 1.1
6 months	72.9 ± 1.0	81.2 ± 1.0*	73.0 ± 1.1	76.7 ± 1.0	79.8 ± 1.1	78.1 ± 1.1	82.3 ± 1.1	77.8 ± 1.0
12 months	73.5 ± 1.0	84.3 ± 1.1*	73.0 ± 1.1	78.2 ± 1.0	80.4 ± 1.2	79.5 ± 1.1	79.5 ± 1.1	81.3 ± 1.1
% Change at 6 months ^C^	−1.9 (−11.0, 7.2)	11.9 (1.3, 22.5)	−6.8 (−32.1, 18.4)	−1.7 (−13.3, 9.8)	5.4 (−15.5, 26.4)	−3.1 (−10.8, 4.7)	5.7 (−7.8, 19.2)	−2.5 (−13.4, 8.2)
% Change at 12 months ^C^	−1.2 (−11.5, 8.9)	15.5 (2.2, 28.2)	−6.8 (−49.0, 36.1)	0.0	6.2 (−17.9, 30.4)	−1.3 (−10.5, 7.8)	2.2 (−14.6,19.1)	1.8 (−11.2, 14.8)
*P*-value (groups) < 0.001; *P*-value (time) = 0.54; *P*-Value (interaction) = 0.002								
**BMI (kg/m**^**2**^**)**								
Baseline	28.5 ± 1.1	30.1 ± 1.1	30.1 ± 1.2	33.4 ± 1.2	31.5 ± 1.0	32.4 ± 1.1	32.9 ± 1.0	32.3 ± 1.0
6 months	28.5 ± 1.1	30.1 ± 1.2	31.1 ± 1.2	35.0 ± 1.2	31.4 ± 1.0	28.2 ± 1.0	30.5 ± 1.0	32.2 ± 1.1
12 months	28.5 ± 1.1	30.1 ± 1.2	-	35.1 ± 1.3	31.6 ± 1.0	30.0 ± 1.1	32.2 ± 1.1	30.6 ± 1.2
% Change at 6 months ^C^	0	0	0	0	0	0	0	0
% Change at 12 months ^C^	0	0	0	0	0	0	0	0
*P*-value (groups) < 0.001; *P*-value (time) = 0.18; *P*-Value (interaction) = 0.36								
**Total Cholesterol (mmol/l)**								
Baseline	4.7 ± 1.0	4.8 ± 1.0	5.3 ± 1.0	5.1 ± 1.0	5.4 ± 1.0	5.3 ± 1.0	4.9 ± 1.0	5.2 ± 1.0
6 months	4.4 ± 1.0	4.4 ± 1.0	5.4 ± 1.1	4.9 ± 1.1	5.1 ± 1.1	5.2 ± 1.0	4.8 ± 1.0	5.1 ± 1.0
12 months	4.1 ± 1.0	4.1 ± 1.0	5.8 ± 1.2	4.6 ± 1.0	4.4 ± 1.2	4.9 ± 1.0	4.5 ± 1.0	5.1 ± 1.0
% Change at 6 months ^C^	−7.0 (−24.8,10.7)	−7.8 (−20.5, 4.7)	2.3 (−28.0, 32.7)	−2.0 (−16.1, 12.1)	−5.6 (−31.1, 19.9)	−0.7 (−10.1, 8.7)	−2.2 (−18.9,14.4)	−1.2 (−13.9, 11.3)
% Change at 12 months ^C^	−2.9 (−7.5, 13.5)	−14.8 (−32.6, 2.9)	10.1 (−29.9, 50.3)	−9.7 (−24.0, 5.4)	−19.5 (−52.5, 13.4)	−6.5 (−17.5, 4.4)	−9.3 (−29.6, 11.0)	−1.6 (−17.8, 14.5)
*P*-value (groups) < 0.001; *P*-value (time) = 0.01; *P*-Value (interaction) = 0.17								
**Triglycerides (mmol/l)**								
Baseline	1.2 ± 0.02	2.4 ± 0.04	1.6 ± 0.07	1.8 ± 0.04	2.5 ± 0.08	1.9 ± 0.03	2.0 ± 0.05	2.0 ± 0.04
6 months	1.2 ± 0.04	1.6 ± 0.04*	1.4 ± 0.13	1.8 ± 0.05	1.9 ± 0.09*	2.1 ± 0.04	1.7 ± 0.06	2.0 ± 0.04
12 months	1.2 ± 0.04	1.5 ± 0.07*	1.7 ± 0.18	1.5 ± 0.06	1.9 ± 0.14	2.0 ± 0.04	1.6 ± 0.09	2.0 ± 0.05
% Change at 6 months ^C^	0	- 42.5 ^a^ (−18.1,-66.8)	−13.1 (−71.2, 45.2)	0	−29.4 ^a^ (−78.7, 19.8)	8.4 (−9.6, 26.6)	−15.0 (47.1, 17.0)	0
% Change at 12 months ^C^	0	−39.2 ^a^ (−5.0, -73.2)	4.0 (−72.3, 82.0)	−9.2 (−65.0, 83.6)	−28.3 ^a^ (−91.0, 35.0)	2.7 (−18.3, 23.8)	−23.4 (−62.4, 15.7)	0
*P*-value (groups) < 0.001; *P*-value (time) = 0.02; *P*-Value (interaction) < 0.001								
**HDL-Cholesterol (mmol/l)**								
Baseline	0.66 ± 0.03	0.95 ± 0.06	1.0 ± 0.09	1.0 ± 0.05	0.91 ± 0.10	0.93 ± 0.03	1.0 ± 0.06	0.94 ± 0.04
6 months	1.0 ± 0.06*	1.0 ± 0.14	0.93 ± 0.28	1.1 ± 0.11	0.90 ± 0.23	0.89 ± 0.07	1.1 ± 0.15	1.0 ± 0.10
12 months	0.98 ± 0.08*	0.98 ± 0.10	-	0.81 ± 0.10	1.0 ± 0.35	0.93 ± 0.09	0.99 ± 0.14	1.0 ± 0.12
% Change at 6 months ^C^	42.7 ^a^ (16.6, 68.9)	8.3 (−49.0,65.8)	−11.3 (−32.6,26.7)	1.3 (−.45.4, 48.4)	1.3 (−63.2, 56.7)	−3.5 (−33.1, 26.0)	11.7 (−51.0, 74.0)	6.4 (−32.6, 45.2)
% Change at 12 months ^C^	37.3 ^a^ (4.3, 70.3)	3.2 (−42.4,48.9)	-	−26.1 (−70.1, 18.6)	12.2 (−14.8, 26.3)	0.0	1.5 (−0.61, 58.3)	8.2 (−43.8, 60.2)
*P*-value (groups) < 0.001; *P*-value (time) = 0.001; *P*-Value (interaction) < 0.001								
**Glucose(mmol/l)**								
Baseline	5.3 ± 1.0	10.0 ± 1.1	7.1 ± 1.3	10.8 ± 1.2	12.4 ± 1.2	10.2 ± 1.0	9.4 ± 1.2	9.1 ± 1.0
6 months	5.7 ± 1.1	9.0 ± 1.0	7.7 ± 1.3	10.3 ± 1.1	10.2 ± 1.1	10.2 ± 1.0	9.1 ± 1.1	9.7 ± 1.0
12 months	5.8 ± 1.1	8.3 ± 1.0	8.2 ± 1.2	9.9 ± 1.0	10.6 ± 1.0	11.2 ± 1.1	9.6 ± 1.2	10.1 ± 1.1
% Change at 6 months ^C^	9.3 (−11.9, 30.6)	−10.5 (−35.1, 14.0)	8.6 (−50.2, 67.5)	1.7 (−29.2, 25.7)	−19.6 (−69.4, 30.0)	0.8 (−17.4, 19.2)	−2.7 (−35.1, 29.6)	7.0 (−17.3, 31.6)
% Change at 12 months ^C^	9.4 (−12.3, 31.2)	−18.2 (−52.8, 16.2)	12.6 (−65.1, 90.7)	−6.7 (−36.3, 22.7)	−15.6 (−79.6, 48.2)	9.7 (−11.2, 31.1)	2.5 (−36.9, 41.9)	10.5 (−19.7, 40.9)
*P*-value (groups) < 0.001; *P*-value (time) = 0.51; *P*-Value (interaction) = 0.26								

Data represented by Mean ± standard error; ^C^ Percent changes represent the absolute difference from baseline in the log-transformed data multiplied by 100; ^b^ p ≤ 0.001; ^a^ p ≤ 0.01; ‘*’ Represents significant difference from the baseline. Level of significance is at p ≤ 0.05.

**Table 2:** Male and female group comparisons of blood pressure and total cholesterol at different time points

**Systolic BP(mmHg)**	**Control**	**Rosiglitazone**	**Diet**	**Insulin**	**Insulin + Orals**	**Metformin**	**Oral agents combination**	**Sulphonylurea**
**Male**								
N	72	48	4	30	6	65	16	26
Baseline	119.1 ± 1.0	129.4 ± 1.0	114.4 ± 1.0	127.1 ± 1.0	139.1 ± 1.1	123.9 ± 1.0	139.3 ± 1.1	123.9 ± 1.0
6 months	131.3 ± 1.0	131.3 ± 1.0	134.0 ± 1.2	125.0 ± 1.0	138.4 ± 1.0	124.1 ± 1.0	132.6 ± 1.0	126.2 ± 1.0
12 months	130.7 ± 1.0	130.6 ± 1.1	-	130.7 ± 1.1	124.0 ± 1.0*	126.2 ± 1.0	133.2 ± 1.1*	128.3 ± 1.0
**Female**								
N	79	1	11	25	6	56	21	31
Baseline	114.0 ± 1.2	139.7 ± 1.0	127.9 ± 1.1	127.6 ± 1.0	121.4 ± 1.0	128.7 ± 1.1	130.4 ± 1.1	127.5 ± 1.0
6 months	115.9 ± 1.1	-	124.6 ± 1.1	122.9 ± 1.0	126.8 ± 1.0	126.2 ± 1.0	124.3 ± 1.0	132.1 ± 1.0
12 months	116.4 ± 1.0	-	132.7 ± 1.0	125.1 ± 1.1	129.1 ± 1.1	129.2 ± 1.0	124.3 ± 1.0*	126.6 ± 1.0
**Diastolic BP(mmHg)**								
**Male**								
Baseline	75.4 ± 1.0	71.8 ± 1.0	72.2 ± 1.0	77.2 ± 1.0	74.5 ± 1.0	80.0 ± 1.0	74.0 ± 1.0	80.0 ± 1.0
6 months	74.4 ± 1.0	80.6 ± 1.1	72.4 ± 1.0	79.0 ± 1.1	80.1 ± 1.1	78.2 ± 1.0	83.0 ± 1.1	83.5 ± 1.1
12 months	74.1 ± 1.0	83.8 ± 1.1*	-	80.7 ± 1.1	87.5 ± 1.1*	76.4 ± 1.0	87.4 ± 1.1*	85.6 ± 1.1
**Female**								
Baseline	74.0 ± 1.0	89.9 ± 1.1	80.4 ± 1.1	80.7 ± 1.1	76.5 ± 1.0	81.2 ± 1.0	80.6 ± 1.0	79.1 ± 1.0
6 months	72.4 ± 1.0	-	74.2 ± 1.0	76.8 ± 1.0	79.7 ± 1.0	78.2 ± 1.0	82.2 ± 1.0	72.5 ± 1.0
12 months	72.2 ± 1.0	-	74.7 ± 1.0	77.4 ± 1.0	77.9 ± 1.0	81.4 ± 1.0	77.3 ± 1.0	78.5 ± 1.0
**Total cholesterol (mmol/l)**								
**Male**								
Baseline	4.4 ± 0.02	4.7 ± 0.02	5.3 ± 0.08	4.9 ± 0.03	5.2 ± 0.06	5.3 ± 0.02	4.7 ± 0.04	5.3 ± 0.03
6 months	4.6 ± 0.02	4.4 ± 0.02	7.2 ± 0.22	5.3 ± 0.04	5.0 ± 0.06	5.1 ± 0.03	4.5 ± 0.05	4.9 ± 0.04
12 months	4.6 ± 0.03	4.0 ± 0.04	8.0 ± 0.22	4.6 ± 0.05	4.4 ± 0.12*	4.9 ± 0.03	4.3 ± 0.08	5.0 ± 0.06
**Female**								
Baseline	4.7 ± 0.02	4.6 ± 0.15	5.3 ± 0.04	5.3 ± 0.04	5.5 ± 0.06	5.2 ± 0.02	5.2 ± 0.03	5.3 ± 0.03
6 months	4.7 ± 0.04	-	5.1 ± 0.06	4.7 ± 0.04	5.1 ± 0.07	5.3 ± 0.02	5.2 ± 0.04	5.4 ± 0.03
12 months	4.7 ± 0.03	-	5.4 ± 0.10	4.4 ± 0.04*	4.4 ± 0.07*	4.9 ± 0.03	4.7 ± 0.05	5.3 ± 0.04

Data represented by Mean ± standard error; ‘*’ Represents significant difference from the baseline. Level of significance is at p ≤ 0.05.

### BMI

The mean BMI of the control group fell within the overweight category, while the mean BMI of the entire DM group fell within the obese-range. No significant change in mean BMI was observed across the follow-ups and this observation even after stratification for gender.

### Blood pressure

There was no significant change in the mean systolic pressure from baseline in any of the groups up to 12 months (Table 1). However, a significant increase was noted in the mean diastolic pressure of the Rosiglitazone group as compared with baseline, while the rest of the groups were similar. After stratifying the groups by gender, we observed a significant decrease from baseline compared with 12-month follow-up in the mean systolic blood pressure among males in the insulin + oral group, as well as males and females in the oral agent-combinations group. Furthermore, there was a significant increase in the mean diastolic blood pressure from baseline compared to the 12-month follow-up among males in the rosiglitazone, insulin + oral and oral agent-combinations groups. This observation was not observed in females, whose mean diastolic blood pressure remained stable along follow-ups.

### Lipid profile

In all groups, there was no significant change noted in the mean cholesterol levels. However, significant improvements were observed in the Rosiglitazone and insulin + orals group in terms of decreased levels of triglycerides, which were not apparent in other groups. Consequently, there was a significant increase in the mean HDL-cholesterol levels in the control group, but not apparent in all DMT2 groups. Stratified by gender, significant improvements were noted in the insulin + orals group in both males and females, as well as the females in the insulin group, in terms of decreased mean total cholesterol. Decreased levels of mean triglycerides were noted in the male subjects under rosiglitazone, insulin + orals and oral agent-combinations, as well as the females in the insulin + orals group. With regards to HDL-cholesterol levels, both males and females in the control group, as well as the males in the insulin + orals group had a significant increase from baseline all the way to the 12-month follow-up (Table 3). The rest of the comparisons were non-significant.

**Table 3:** Male and female group comparisons of hdl-cholesterol, triglycerides and vitamin d at different time points

**HDL(mmol/l)**	**Control**	**Rosiglitazone**	**Diet**	**Insulin**	**Insulin + Orals**	**Metformin**	**Oral agents combination**	**Sulphonylurea**
**Male**								
Baseline	0.57 ± 0.05	0.94 ± 0.06	0.99 ± 0.22	0.94 ± 0.08	0.81 ± 0.18	0.92 ± 0.06	0.93 ± 0.11	0.92 ± 0.09
6 months	0.92 ± 0.09*	1.0 ± 0.17	-	1.0 ± 0.17	0.86 ± 0.26	0.81 ± 0.13	1.1 ± 0.26	1.0 ± 0.17
12 months	0.94 ± 0.15*	0.97 ± 0.13	-	0.88 ± 0.12	1.1 ± 0.32*	0.72 ± 0.19	0.88 ± 0.30	0.98 ± 0.28
**Female**								
Baseline	0.76 ± 0.03	0.88 ± 0.22	1.1 ± 0.07	1.1 ± 0.04	1.0 ± 0.09	0.96 ± 0.03	1.0 ± 0.05	0.99 ± 0.04
6 months	1.0 ± 0.06*	-	1.0 ± 0.16	1.0 ± 0.13	1.0 ± 0.15	1.0 ± 0.05	1.2 ± 0.13	1.0 ± 0.07
12 months	0.99 ± 0.06*	-		1.2 ± 0.08	1.0 ± 0.17	1.1 ± 0.07	1.1 ± 0.10	1.1 ± 0.09
**Triglycerides (mmol/l)**								
**Male**								
Baseline	1.3 ± 0.03	2.3 ± 0.04	1.6 ± 0.16	1.7 ± 0.06	2.7 ± 0.13	2.1 ± 0.04	2.3 ± 0.08	2.2 ± 0.06
6 months	1.2 ± 0.06	1.5 ± 0.04*	1.1 ± 0.21	1.9 ± 0.09	1.7 ± 0.13*	2.4 ± 0.07	1.6 ± 0.09*	1.8 ± 0.07
12 months	1.4 ± 0.07	1.5 ± 0.07*	1.3 ± 0.22	1.7 ± 0.10	2.2 ± 0.12	2.3 ± 0.07	1.5 ± 0.12*	2.0 ± 0.11
**Female**								
Baseline	1.2 ± 0.03	2.5 ± 0.28	1.6 ± 0.08	1.9 ± 0.05	2.1 ± 0.11	1.7 ± 0.04	1.8 ± 0.06	1.9 ± 0.05
6 months	1.3 ± 0.08	-	1.5 ± 0.12	1.8 ± 0.07	2.1 ± 0.13	1.8 ± 0.05	1.9 ± 0.09	2.2 ± 0.06
12 months	1.2 ± 0.06	-	1.8 ± 0.20	1.3 ± 0.08	1.7 ± 0.13*	1.6 ± 0.06	1.6 ± 0.10	2.0 ± 0.07
**Vitamin D (nmol/l)**								
**Male**								
Baseline	21.6 ± 1.0	33.7 ± 1.0	25.3 ± 1.0	34.9 ± 1.0	35.1 ± 1.0	35.7 ± 1.0	34.5 ± 1.0	34.5 ± 1.0
6 months	30.8 ± 1.0*	48.4 ± 1.0*	39.1 ± 1.1*	57.6 ± 1.1*	52.8 ± 1.2*	60.6 ± 1.1*	49.6 ± 1.1*	49.6 ± 1.1*
12 months	30.2 ± 1.1*	50.4 ± 1.1*	23.1 ± 1.0	50.1 ± 1.1*	45.8 ± 1.1*	54.5 ± 1.0*	53.7 ± 1.0*	53.1 ± .1.1*
**Female**								
Baseline	17.6 ± 1.0	19.2 ± 1.0	24.0 ± 1.0	29.6 ± 1.0	33.2 ± 1.0	34.7 ± 1.0	34.6 ± 1.1	40.4 ± 1.0
6 months	24.2 ± 1.0	-	33.3 ± 1.1	53.5 ± 1.1*	59.1 ± 1.0*	66.0 ± 1.2*	42.1 ± 1.2*	54.5 ± 1.1*
12 months	22.6 ± 1.0	-	29.2 ± 1.0	45.4 ± 1.1*	49.1 ± 1.0*	57.9 ± 1.1*	42.9 ± 1.2*	46.8 ± 1.0*

Data represented by Mean ± standard error; ‘*’ Represents significant difference from the baseline. Level of significance is at p ≤ 0.05.

## Discussion

We have already demonstrated from previous cross-sectional observations that non-diabetic adults are more vitamin D deficient than DMT2 adults, probably because of improved diet and multi-vitamin supplementation [10]. We have also shown that vitamin D supplementation even up to 18 months is not sufficient to fully correct vitamin D status, but nevertheless confers improvement in the metabolic profiles among the DMT2 adults, including insulin sensitivity [7]. The significant improvements in the metabolic status of DMT2 subjects secondary to vitamin D supplementation have also been documented in other interventional and clinical trial studies with respect to glucose homeostasis, although beneficial effects in vascular function fell short [11,12]. A recent review by Pilz and colleagues observed that the modest effects of vitamin D on glycemic control and insulin resistance in a few randomized controlled trials are insufficient to recommend vitamin D supplementation for DMT2 patients [13]. The present interventional study highlighted how the different anti-diabetic therapies influence/interfere with circulating levels of 25-OHVitD before and after 12-months of vitamin D supplementation, and shed light as to how these anti-DM therapies inhibit/activate the beneficial effects of such intervention.

In the rosiglitazone group, vitamin D supplementation increased the mean circulating 25-OHVitD level of all subjects and had favorable effects on the circulating triglycerides, which was observed only in males. Current evidence states that rosiglitazone is not superior to other anti-diabetic drugs, such as metformin, when it comes to improving lipid profile [14]. Nevertheless, rosiglitazone affects vitamin D status by selective agonism of peroxisome proliferator activated receptors (PPAR-gamma), which are present in muscle, liver and adipose tissue [15]. It is probably through the agonist effect of rosiglitazone on circulating 25-OHVitD levels that indirectly contributed to the parallel improvement of triglycerides, at least in the male group since no information was available in females.

In the insulin alone group, significant improvement was observed in the mean total cholesterol of females, while other metabolic parameters remained unchanged. The finding is in accordance with a recent clinical trial done in females with polycystic ovary syndrome (PCOS), who also benefitted from vitamin D3 supplementation in terms of decreased cholesterol levels [16]. However, the improvement in the cholesterol levels secondary to vitamin D correction were not observed in other trials involving the general population in both the short- and long term [17,18].

In contrast, the insulin + oral agents group showed more significant improvements in the metabolic profile, which included triglycerides and total cholesterol, as well as systolic blood pressure and HDL-cholesterol in the males. Vitamin D correction may protect against increased cardiovascular risk through the promotion of large HDL particle formation, which increases reverse cholesterol transport [15]. These large HDL-cholesterol particles may form because of stimulation of apolipoprotein A1, the largest component of HDL-cholesterol, by vitamin D [19]. While the mechanism of why this effect was only apparent in the insulin + oral agents group cannot be addressed in the present study, it is worthy to mention that vitamin D correction under this particular regimen was more beneficial in terms of improving cardiometabolic profile than the standard DMT2 treatments.

No significant changes were seen in other groups (metformin, oral agent-combinations and sulphonylurea) in relation to vitamin D supplementation, with the exception of increased levels of 25-OHVitD. Of note is the lack of a significant increase in the HDL-cholesterol levels of the sulphonylurea group, which can be clinically favorable, since DM patients on sulphonylureas had a lower HDL-cholesterol than DMT2 patients on insulin and diet, not to mention the lower ability of sulphonylureas to improve the lipid profile, making it a positive risk factor for ischemic heart disease in patients with DMT2 [20-23]. It is also clinically favorable in the sense that most probably the vitamin D supplementation is sufficient to counteract the HDL-lowering ability of sulphonylureas, but not sufficient in increasing HDL-levels, making the final level similar to previous measurements. Furthermore, the increased 25-OHVitD levels in the metformin group with no added metabolic improvements may imply, at least, that metformin does not affect negatively vitamin D correction in these patients [24].

The authors acknowledge several limitations. The small sample size of some DMT2 groups may have created bias and thus results from these groups should be interpreted with caution. Other confounders, such as diet and physical activity, were not accounted for in the study. Furthermore the mean doses of medications were not documented and therefore the dose dependent effect cannot be verified. Nevertheless, this is one of the first studies to compare how different DMT2 therapies affect vitamin D supplementation. While all groups seemed to increase their levels of 25-OHVitD after onset supplementation, it appears that those DMT2 patients on insulin in combination with other drugs was the group that benefitted the most as compared with other groups in terms of improving cardiovascular risk. Further studies on the effects of vitamin D supplementation in harder outcomes such as HOMA β-function in these groups should be considered.

## Competing interests

The authors have no conflict of interest to disclose related to this study.

## Authors’ contributions

KMA and NMA conceived the study, oversaw study execution, and contributed to manuscript writing. AA and OM recruited subjects and collected data. MSA and YA recruited subjects and analyzed samples. SS and GPC reviewed data analysis and wrote the manuscript. SK and GPC reviewed/edited the final version of the manuscript. All authors read and approved the final manuscript.
